# Body Dysmorphic Disorder (BDD) and Social Anxiety Disorder (SAD) in Urban Indian Medical Students: A Cross-Sectional Study

**DOI:** 10.7759/cureus.90559

**Published:** 2025-08-20

**Authors:** Amalesh S Honnekeri, Bindoo Jadhav, Navoneela Bardhan

**Affiliations:** 1 Psychiatry, K.J. Somaiya Medical College, Mumbai, IND

**Keywords:** body dysmorphic disorder, medical education, medical students, screening, social anxiety disorder, urban india

## Abstract

Background

Body dysmorphic disorder (BDD) is characterized by a distorted self-image, which causes patients anxiety and excessive worry about their appearance. Social anxiety disorder (SAD) is characterized by a fear of embarrassment in social situations. This study aimed to screen for BDD and examine its prevalence and relationship with SAD among urban Indian medical students.

Methods

A total of 176 medical students from 4 medical colleges in Mumbai were screened. They were administered the BDD Questionnaire, the Social Interaction Anxiety Scale, and the Social Phobia Scale, which were then compared for analysis using the chi-square test, Student’s t-test, and Spearman’s rho.

Results

The overall prevalence of BDD in urban Indian medical students was found to be 30.7%. There was no significant difference in the prevalence of BDD between males (33.3%) and females (29.3%) (chi-square = 0.301, df = 1, p = 0.583). BDD scores positively correlated with SIAS scores (Spearman's rho = 0.242, p=0.001), implying a significant association between BDD and SAD.

Conclusions

Medical students with BDD also reported having SAD, implying that a distorted self-image and worry about appearance also significantly hinder social communication and behaviour. Given the significant association between BDD and SAD, clinicians should consider screening for SAD in patients with BDD, and vice versa, to enable early intervention and integrated treatment.

## Introduction

Body dysmorphic disorder (BDD) is a chronic, debilitating condition characterized by a preoccupation with perceived defects or flaws in physical appearance. These defects, however, may not be apparent to others [1]. The Diagnostic and Statistical Manual of Mental Disorders (DSM-5) has classified BDD in the category of obsessive-compulsive and related disorders [2]. Associated comorbidities of BDD include impairment in normal-functioning depression and suicidality [3,4].

BDD remains underdiagnosed, and patients have an overwhelming preoccupation with self-perceived defects in their appearance, which may not even be apparent to external onlookers. Their preoccupation causes them unusually high psychological distress, which may affect their daily functioning as they fixate on their appearance [5].

Numerous studies have been conducted on the prevalence of BDD in multiple settings, such as in adolescents, students, psychiatric inpatients and outpatients, patients of cosmetic surgery, patients of rhinoplasty surgery, and cosmetology outpatients [6].

A cross-cultural study assessing BDD in a group of 101 American students found its prevalence to be 4% [7]. Compared to a sample of 133 German students, American students reported significantly higher levels of body image concerns and preoccupation.

In India, a study conducted in a Dermatology outpatient setting found the prevalence of BDD to be 7.5% in patients with cosmetic complaints, while in patients with general dermatologic complaints, it was found to be 2.1% [8]. However, there was no significant difference in these rates.

While the prevalence of BDD hasn’t yet been established in Indian medical students, several attempts have been made globally to shed light on this condition in similar cohorts. For instance, in a study conducted on medical students in Karachi, Pakistan, the prevalence of BDD was assessed to be 5.8% [9].

Social anxiety disorder (SAD) is a condition wherein a person has an intense fear of social situations, as he or she may anticipate being negatively evaluated [10]. The DSM-5 has classified SAD as an anxiety disorder [11]. Studies have shown that SAD has the potential to substantially increase the risk of depression, suicidality, substance abuse, lower work and educational attainment, and victimization [12].

In India, a study conducted in a government medical college in the state of Gujarat found the prevalence of SAD in medical students to be 11.37% [13]. Another study conducted in an urban Indian setting found the prevalence of SAD in undergraduate medical students to be 7.8% [14]. This study proposed Internet-based cognitive behavioral therapy (ICBT) as a potential line of treatment for patients with SAD, who were found to prefer online communication rather than face-to-face interaction.

There has already been established evidence to suggest that BDD and SAD are comorbid, with a study revealing that improvements in one of the two disorders significantly correlated with improvements in the other, on treatment [15]. Therefore, it was thought appropriate to further explore this relationship in urban Indian medical students, a study population that happens to face psychological stress and a hectic lifestyle, which negatively affects their mental well-being [16].

This study is thus a step toward bridging the existing gap in knowledge regarding the prevalence of BDD in a study population that is otherwise highly productive and competent. This study aims to screen for the presence of BDD, identify gender-based patterns in body image concerns, and explore its relationship with SAD among urban Indian medical students.

This study was selected for oral presentation at the Leiden International (Bio)Medical Student Conference (LIMSC) held at the University of Leiden, Leiden, Netherlands, in March 2019.

## Materials and methods

Objectives

This study aimed to screen for the presence of BDD and examine its association with SAD among urban Indian medical students.

Study type, site, and participants

This was a prospective cross-sectional study conducted across four medical colleges in Mumbai, India. Participants were recruited using convenience sampling from medical students who met the inclusion criteria and provided informed consent. A total of 250 medical students were potentially eligible and administered the study materials, of which 176 consented to participate and filled out the questionnaires (response rate: 70.4%).

The inclusion criteria were: a. Participants should be medical students residing in Mumbai, India; b. Participants should be 18 years of age or above; c. Participants should have a working knowledge of English

Medical students who were below 18 years of age, did not have a working knowledge of English, or did not reside in Mumbai were excluded from the study.

Ethical considerations

Institutional Ethics Committee approval was obtained prior to the commencement of this study, which adhered to the ‘Declaration of Helsinki’. Participants were provided with an information document detailing the purpose of this study, declaring their participation as voluntary and not affecting any ongoing treatment they may be on, and explaining potential risks and benefits to their participation. Additionally, anonymity and confidentiality were assured to all study participants.

Study materials

The following materials were administered to participants: a demographic questionnaire containing the participant’s age, gender, current year of medical education, the BDD Questionnaire (BDDQ; Phillips et al.) [7], the Social Interaction Anxiety Scale (SIAS; Mattick and Clarke), the Social Phobia Scale (SPS; Mattick and Clarke) [13], and an informed consent form with a participant information sheet. Requisite permissions were obtained for the use of these instruments.

The BDDQ is a self-reported questionnaire, used as an efficient first step to identify patients who have BDD, especially in larger cohorts. It contains a total of four objective questions, and their responses (“Yes” or “No”) indicate the likelihood of BDD.

The SIAS and SPS are both 5-point Likert scales that are self-reported. Each item is scored from 0 ('not at all characteristic or true of me') to 4 ('extremely characteristic or true of me'). Both scales assess the presence of social anxiety disorders.

Data collection methods

Participants were recruited by the student-researcher directly and by word-of-mouth publicity, after taking requisite permissions from the concerned authorities. Participants placed completed, anonymous surveys in sealed envelopes and returned them to the researcher during college hours to ensure confidentiality. The data collected were then imported into Microsoft Excel spreadsheets (Microsoft Corporation, Redmond, WA, US) for biostatistical analysis.

Data analysis

Categorical data was compared using the chi-square test, and quantitative data was compared using the Student's t-test. Categorical data were described using counts and percentages, and quantitative data were described using mean +/- SD. For correlation, Spearman's rho was used.

## Results

Prevalence of BDD in the study sample

Among the study population, BDD was found to be present in 54 (30.7%) respondents.

Gender-wise prevalence of BDD

Of the 176 respondents, 116 were female and 60 were male. Thirty-four (29.3%) female respondents and 20 (33.2%) male respondents reported having BDD. On comparison via the chi-square test, there was no significant difference in the proportion of BDD among male and female respondents (Table 1).

**Table 1:** Gender differences in the prevalence of BDD in the study sample chi-square = 0.301, df = 1, p = 0.583 BDD: body dysmorphic disorder

		BDD PRESENT		Total
		No	Yes	
GENDER	Female	82 (70.7%)	34 (29.3%)	116 (100.0%)
	Male	40 (66.7%)	20 (33.3%)	60 (100.0%)
Total		122 (69.3%)	54 (30.7%)	176 (100.0%)

In females who tested positive for BDD, the major body part of concern was found to be the chest, as reported by 11 respondents (32.4%), while in males who tested positive for BDD, the major body part of concern was the arm, as reported by 6 respondents (30.0%).

Prevalence of SAD in the study sample

In the study sample, SAD, as tabulated through the SIAS, was found to be present in 26 respondents (14.8%).

Prevalence of non-generalized social phobia in the study sample

In the study sample, non-generalized social phobia, as tabulated through the SPS, was found to be present in 17 respondents (9.7%).

Relationship between BDD and SAD

**Figure 1:**
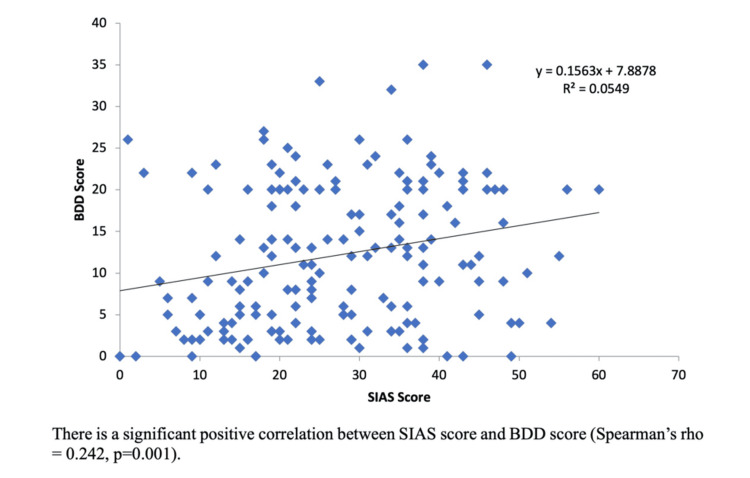
The relationship between BDD and SAD There is a significant positive correlation between the SIAS score and the BDD score (Spearman's rho = 0.242, p=0.001), implying a positive correlation between BDD and SAD. BDD: body dysmorphic disorder; SAD: social anxiety disorder; SIAS: Social Interaction Anxiety Scale

A significant positive association was observed between BDD and SAD (Figure 1).

## Discussion

The overall presence of BDD in the cohort of urban Indian medical students that was screened was found to be 30.7%. BDD in this population does not seem to have been studied on a prior occasion. Thus, this study has established a baseline presence of BDD in this specific population group.

The observed prevalence (30.7%) exceeds rates reported among medical students in Pakistan (5.8%) [9] and general student populations in the US (4%) [7].

Even within India, the presence of BDD in this group appears to be higher than in other study groups; for example, it was 7.5% in patients with cosmetic complaints and 2.1% in patients with general dermatologic complaints in an Indian dermatology outpatient setting [8].

There can be several possible explanations for this, including a difference in lifestyle, high amounts of psychological stress, and lower mental well-being among Indian medical students, as mentioned previously [9,13,14,16]. A potential future direction for studies that build on the information obtained in this study could be to take the academic schedule of Indian medical students into consideration, along with the various curriculum changes that occur in the MBBS course. The daily lifestyle of these students needs to be assessed in depth to further understand if there is any correlation between hours available for grooming and body dysmorphia.

Further, it is imperative to note that the cohort that participated in this study resided in Mumbai, an urban metropolis, which is also home to Bollywood and Indian pop culture and is a large and populous city [17]. The high prevalence of BDD found in this study points toward the possibility of urban culture influencing body image and perception, leading to a tendency to develop obsessive-compulsive thought processes.

The presence of BDD in male respondents was found to be 33.3%, while in female respondents, it was found to be 29.3%. It was observed that there was no significant difference in the proportion of BDD between males and females (chi-square = 0.301, df=1, p=0.583). The study conducted among medical students in Karachi, Pakistan, revealed that there was a significantly higher prevalence of BDD in males than in females, with a prevalence ratio of 1.7 [9], which opposes the findings of this study. These findings suggest that gender does not significantly influence BDD prevalence in this cohort, supporting the need for gender-neutral screening approaches and also implying that body image issues may have more intricate root causes that need to be further explored in subsequent studies. It is also imperative to mention that this study did not take into account gender identities outside the binary spectrum, which is another direction further studies should explore.

In females who were found to be positive for BDD, the major body part of concern was the chest (32.4%), while in males who were positive for BDD, the major body part of concern was the arm (30.0%). In the previously mentioned study conducted in Karachi, the major focus of concern in female students was body fat (40.4%), while in males it was head hair (34.3%) [9]. These differences may possibly be due to different cultural influences that are prevalent in the two study groups; therefore, we recommend cross-cultural studies to further understand the reasons behind this finding from our study.

Potential factors worth assessing in future studies that might be contributing to these findings include the differences in ethnic garments worn across cultures, distinct cultural values, differing pop culture influences, and the role of media, celebrities, and both contemporary and traditional cultural values in shaping body image perception. From our study, we can conclude that these perceptions are dynamic, as they differ in different settings.

Healthcare providers, including psychiatrists and psychotherapists, should consider sociocultural factors that influence body image and mental health. This information is also useful in cosmetology clinics, as healthcare providers who work in these clinics may be able to use this data to identify potential body dysmorphia and liaise with mental healthcare professionals to help these patients.

The prevalence of SAD in this study group was found to be 14.8%. Another study assessing SAD in urban Indian medical students had found its prevalence to be 7.8% [14], while a study conducted in Indian high school students had found it to be 12.8% [18]. In this study, 9.7% of the sample were found to have non-generalized social phobia. In another study conducted among urban Indian medical students, the prevalence of non-generalized social phobia was found to be 23% [14], while a study conducted in a rural Indian setting found 4.8% of the adolescents surveyed to have non-generalized social phobia [19]. This indicates that the rates of social anxiety and social phobia in urban Indian medical students lie within a similar range as the rates of social anxiety and social phobia in the general population.

On comparison of BDD scores with SIAS scores obtained in the study, it was found that there was a positive correlation between the two (Spearman’s rho = 0.242, p = 0.001), implying a significant association between BDD and SAD. This association implies that a distorted self-image and worry about appearance might significantly hinder social communication and behaviour. This can negatively impact quality of life. Given this relationship, healthcare providers may consider screening for the other when one is detected. Additionally, the potential of body image issues leading to the development of eating disorders [20] also warrants screening for the latter in individuals susceptible to BDD.

Notably, the preferred first-line treatment for both BDD and SAD is cognitive behavioral therapy (CBT) [21,22]. Pharmacologically, selective serotonin reuptake inhibitors (SSRIs) are the drug of choice in the treatment of both disorders [23,24]. It may be prudent to combine the treatment for both disorders when they are present together in an individual. This will not only help cope with BDD but also enable better socialization and improve quality of life.

Lastly, given the high prevalence of both BDD and SAD in this cohort, healthcare training institutions and medical schools may consider collaborating with psychologists and psychiatrists to formulate mental healthcare resources for medical students that not only target anxiety management, but also identify and deal with disorders on the obsessive-compulsive spectrum, which are also present in greater amounts in this cohort as compared to the general population [7,8].

Limitations of the study

This study analyzed the gender-wise presence of BDD and compared it to previous data wherein gender was measured as a binary. A future direction for this study and similar studies could be to analyze BDD as a disorder among various gender and sexual identities outside the binary spectrum. Additionally, this study relied on data that was self-reported, and self-reporting bias might contribute to study constraints. Further, the use of convenience sampling might also contribute to study constraints due to the possibility of selection bias. 

## Conclusions

Medical students with BDD were more likely to report SAD, suggesting that distorted self-image and appearance-related concerns may impair social communication and behavior. Given this relationship, healthcare providers may consider screening for the other when one is detected. Healthcare providers can also try to devise comprehensive therapeutic strategies that target both disorders simultaneously, thus not only helping cope with BDD but also enabling better socialization and improving quality of life. These findings represent significant advancements in understanding the socio-cultural epidemiology of both BDD and SAD in a population that is otherwise considered to be highly productive - medical students. This study has also established the need to further assess BDD as a disorder among populations similar to this cohort, to gain a more in-depth understanding of its causes, triggering circumstances and situations, and long-term effects.

## References

[1] Thungana Y, Moxley K, Lachman A (2018). Body dysmorphic disorder: a diagnostic challenge in adolescence. S Afr J Psychiatr.

[2] Schieber K, Kollei I, de Zwaan M, Martin A (2015). Classification of body dysmorphic disorder - what is the advantage of the new DSM-5 criteria?. J Psychosom Res.

[3] França K, Roccia MG, Castillo D (2017). Body dysmorphic disorder: history and curiosities. Wien Med Wochenschr.

[4] Malcolm A, Labuschagne I, Castle D, Terrett G, Rendell PG, Rossell SL (2018). The relationship between body dysmorphic disorder and obsessive-compulsive disorder: a systematic review of direct comparative studies. Aust N Z J Psychiatry.

[5] Varma A, Rastogi R (2015). Recognizing body dysmorphic disorder (dysmorphophobia). J Cutan Aesthet Surg.

[6] Veale D, Gledhill LJ, Christodoulou P, Hodsoll J (2016). Body dysmorphic disorder in different settings: a systematic review and estimated weighted prevalence. Body Image.

[7] Bohne A, Keuthen NJ, Wilhelm S, Deckersbach T, Jenike MA (2002). Prevalence of symptoms of body dysmorphic disorder and its correlates: a cross-cultural comparison. Psychosomatics.

[8] Thanveer F, Khunger N (2016). Screening for body dysmorphic disorder in a dermatology outpatient setting at a tertiary care centre. J Cutan Aesthet Surg.

[9] Taqui AM, Shaikh M, Gowani SA (2008). Body dysmorphic disorder: gender differences and prevalence in a Pakistani medical student population. BMC Psychiatry.

[10] Leichsenring F, Leweke F (2017). Social anxiety disorder. N Engl J Med.

[11] (2016). DSM-5 Changes: Implications for Child Serious Emotional Disturbance [Internet]. https://www.ncbi.nlm.nih.gov/books/NBK519712/.

[12] Garcia-Lopez LJ, Bonilla N, Muela-Martinez JA (2016). Considering comorbidity in adolescents with social anxiety disorder. Psychiatry Investig.

[13] Ratnani IJ, Vala AU, Panchal BN, Tiwari DS, Karambelkar SS, Sojitra MG, Nagori NN (2017). Association of social anxiety disorder with depression and quality of life among medical undergraduate students. J Family Med Prim Care.

[14] Honnekeri BS, Goel A, Umate M, Shah N, De Sousa A (2017). Social anxiety and Internet socialization in Indian undergraduate students: an exploratory study. Asian J Psychiatr.

[15] Fang A, Hofmann SG (2010). Relationship between social anxiety disorder and body dysmorphic disorder. Clin Psychol Rev.

[16] Nandi M, Hazra A, Sarkar S, Mondal R, Ghosal MK (2012). Stress and its risk factors in medical students: an observational study from a medical college in India. Indian J Med Sci.

[17] Akhuly A, Kulkarni M (2010). Public mental health services in Mumbai. Int Psychiatry.

[18] Mehtalia K, Vankar GK (2004). Social anxiety in adolescents. Indian J Psychiatry.

[19] Nair MK, Russell PS, Subramaniam VS (2013). ADad 8: School Phobia and Anxiety Disorders among adolescents in a rural community population in India. Indian J Pediatr.

[20] Himmerich H, Mirzaei K (2024). Body image, nutrition, and mental health. Nutrients.

[21] Hofmann SG (2007). Cognitive factors that maintain social anxiety disorder: a comprehensive model and its treatment implications. Cogn Behav Ther.

[22] Hofmann SG, Smits JA (2008). Cognitive-behavioral therapy for adult anxiety disorders: a meta-analysis of randomized placebo-controlled trials. J Clin Psychiatry.

[23] Roy-Byrne PP, Cowley DS (2007). Pharmacological treatments for panic disorder, generalized anxiety disorder, specific phobia, and social anxiety disorder. A Guide to Treatments That Work.

[24] Williams J, Hadjistavropoulos T, Sharpe D (2006). A meta-analysis of psychological and pharmacological treatments for body dysmorphic disorder. Behav Res Ther.

